# Afforestation using a range of tree species, in New Zealand: New Forest trial series establishment, site description, and initial data

**DOI:** 10.1016/j.dib.2024.110321

**Published:** 2024-03-12

**Authors:** Thomas S.H. Paul, Loretta G. Garrett, Simeon J. Smaill

**Affiliations:** a Scion, Private Bag 3020, Rotorua 3046, New Zealand; b Scion, PO Box 29237, Riccarton, Christchurch 8440, New Zealand

**Keywords:** Planted forests, Productivity, Non-timber benefits, Carbon

## Abstract

Global climate change and shift towards a bio-economy has heightened the need to design sustainable forestry systems that balance economic, environmental and social benefits. In New Zealand, production forests are dominated by planted Pinus radiata, which makes up 90 % of the planted forest area. There is very little data driven evidence in New Zealand to support diversifying across a range of tree species and what timber and non-timber benefits may be gained by diversifying tree species in New Zealand's production forests. The New Zealand New Forest Trial Series (NFTS) was designed and established in 2013 on marginal pastoral land to address the knowledge gap for how afforesting land with different trees species, both exotic and indigenous to New Zealand, across a climate range can deliver to both timber and non-timber benefits. These trials were planted with *Cupressocyparis ovensii, Eucalyptus fastigata, Fraxinus excelsior, Nothofagus fusca (*plus *Leptospermum scoparium*), *Pinus radiata, Podocarpus totara* and *Sequoia sempervirens* plus a control with no planting to monitor natural succession. The Before-After-Control-Impact (BACI) experiment design has collected pre-planting data describing the present vegetation and a range of soil properties, presented in this paper. This will allow the comparative monitoring of the changes that will occur through planting the various tree species on marginal land in different environments through time. With time the long-term trials will deliver data evidence on tree species survival when planted into marginal pastoral land, tree productivity and the flow of economic, environmental and social benefits from the new plantings. This knowledge will strengthen New Zealand's forestry sector confidence to make informed decisions to diversify tree species with changing climatic and social challenges.

Specifications Table

**Table:** 

Subject	Forestry
Specific subject area	Establishment of new forests with a range of tree species to demonstrate timber and non-timber benefits.
Data format	Raw
Type of data	Tables and images.
Data collection	Data were collected from field measurements and laboratory analysis of field collected soil samples. Plot slope was measured using a clinometer, aspect using compass and altitude using a GPS. Plant diversity was collected from each plot in the field using height tiers and percent cover. Soil properties were collection from in field observations at the centre of each plot to record soil horizon depth and horizon notation down to 1 m depth, and topsoil depth. Also collected from the plot centre were soil bulk density samples collected by depth increment (0–10, 10–20, 20–30 cm) and intact cores for porosity testing collected within the 0–10 cm depth increment. Soil samples collected by depth increment (0–10, 10–20, 20–30 cm) from a grid pattern over the plot were tested for total carbon and total nitrogen using a LECO FPS-21000 CNS thermal combustion furnace and diffuse reflectance mid-infrared spectra were acquired using a Bruker Invenio-S Fourier transform infrared spectrometer with a Bruker HTS-XT (High Throughput Screening Extension) microplate reader fitted with a liquid nitrogen cooled mercury cadmium telluride detector. The 0–10 cm soil sample collected from each plot using a grid pattern was also used to examine the diversity in carbon substrate utilisation by the soil microbial population.
Data source location	Scion, New Zealand. Location of the four trials, latitude and longitude, are in data tables.
Data accessibility	Repository name: Figshare Data identification number: (or DOI or persistent identifier) https://doi.org/10.6084/m9.figshare.23605746.v1
Related research article	N/A

## 1 Value of the Data

• The data provides a baseline to New Zealand's New Forest Trial Series which can then be used to compare all other data collected from these trials. Baseline data for afforestation studies in New Zealand is rare and will contribute towards understanding the effects of land use change.
• The data originates from four sites that represent large areas in New Zealand that are targeted for afforestation, because of their low pastoral productivity (marginal hill country).
• The data can be used to determine soil carbon and nutrient pools, plant diversity and soil microbial activity giving the starting conditions at time of planting.
• The trial infrastructure and starting data conditions will benefit New Zealand and global forestry science as data is collected over time targeted towards afforestation establishment, and timber and non-timber benefits of a variety of tree species.

## 2 Background

The compiled dataset is the initial dataset of a long-term trial series that aims to understand the effects of different trees species on the provision of economic (timber production) and environmental services on afforestation sites in New Zealand. With increasing calls for diversification of planted forests and a wider provision of multiple services through forests the need for quantitative data motivated the installation of these trials. The creation of baseline data will allow us to track the ecosystem changes occurring on these sites through long-term monitoring and at the same time provides vegetation, soil and microbial activity data for sites that are chosen to be afforested by New Zealand landowners.

## 3 Data Description

**Fig. 1:**
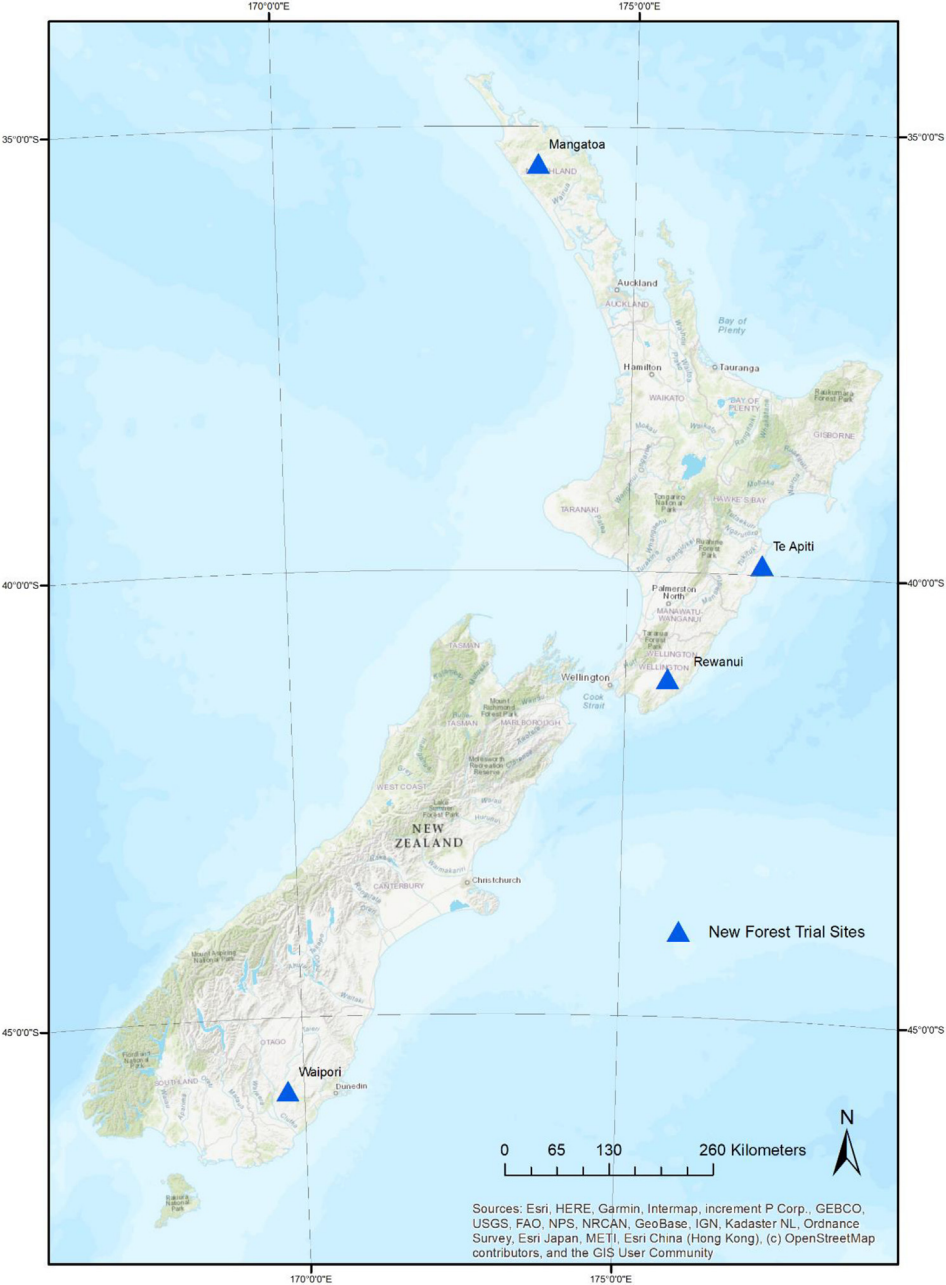
Location of the four New Forest Trial Sites within New Zealand.

The dataset contains raw data collected from four New Forest trials in New Zealand. Table 1 gives a description for each of the trial site, Table 2 gives a list of the tree species planted, and Fig. 1 shows the spatial distribution of the four New Forest trials within New Zealand.

**Table 1:** Site description.

Variable	Site			
Trial ID*	FR531/1	FR531/2	FR531/3	FR531/4
Site	Mangatoa	Te Apiti	Rewanui	Waipori
Latitude (°)	−35.42	−39.86	−40.93	−45.80
Longitude (°)	173.64	176.96	175.92	169.80
Elevation (m asl)	95	134	200	695
Mean air temperature ( °C) [3]⁎⁎	15.3	12.9	12	7
Mean annual relative air humidity (%)⁎⁎	86	78	80	82
Mean annual rainfall (mm) [3]⁎⁎	1551	1136	1024	850
Slope (degrees)	8	17	0	10
Soil drainage	Imperfect	Imperfect to moderate	Imperfect to well	Imperfect
New Zealand Soil Classification [4]	Mottled Yellow Ultic	Mottled Firm Brown and Pallic Firm Brown	Mottled Orthic Brown and Pallic Orthic Brown	Mottled Orthic Brown

⁎ Trial ID is an individual trial identification allocated to a trial site by Scion's (New Zealand Forest Research Institute Ltd) forestry trials database.

⁎⁎ Climate data normalised 1980–2010.

**Table 2:** List of tree species planted in the trial series.

Tree species name	Tree species common name	Native or exotic species to New Zealand	Planting stocking
*Cupressocyparis ovensii*	Cypress	Non-native	2.2 m x 2.2 m (∼2000 stems/ha)
*Eucalyptus fastigata*	Eucalyptus	Non-native	
*Podocarpus totara*	Totara	Native	
*Sequoia sempervirens*	Redwood	Non-native	
Nothofagus fusca*	Red Beech	Native	
*Fraxinus excelsior*	Ash	Non-native	2.8 m x 2.8 m (∼1300 stems/ha)
*Pinus radiata*	Radiata pine	Non-native	
Control unplanted	—-	—–	—–

⁎ Due to early mortality of red beech, the species was replaced (at Mangatoa and Rewanui) and interplanted (at Te Apiti) with mānuka (*Leptospermum scoparium*) in the affected plots in 2015.

The raw data on initial (time zero) soil, plant, and trial details for the New Forest trials series (FR531) in New Zealand are stored in Figshare [1].

## 4 Experimental Design, Materials and Methods

### 4.1 Site selection and description

Four New Forest trials were installed in New Zealand on marginal land with low pastoral productivity [2] and in different environments in New Zealand, site details in Table 1.

### 4.2 Trial design

The trial was designed as a Before-After-Control-Impact (BACI) experiment to allow for comparative monitoring of the changes that will occur through planting the various tree species on marginal land in different environments through time. Each treatment of planting a specific tree species or natural succession (control) (Table 2) was replicated three times in a block design. Square plots 50×50 m were installed with a 0.04 ha circular measurement plot. At the centre of the measurement plot, slope was measured using a clinometer, aspect using a compass, altitude measured using a GPS, and landform physiography and slope shape were described.

### 4.3 Species selection and planting

Seven treatments of planting a specific tree species (Table 2) in 50×50 m square plots, containing a 0.04 ha circular measurement plot were installed on each site in the winter of 2013, replicated three times. The New Zealand native tree species with productive potential (*Podocarpus totara* G.Benn.ex.D.Don, *Nothofagus fusca* (Hook.f.) Oerst.) as well as non-native hardwoods and conifers (*Eucalyptus fastigata* H.Deane & Maiden, *Fraxinus excelsior* L., *Sequoia sempervirens* D.Don, *Cupressocyparis ovensii* A.F.Mitch), and New Zealand's most commonly planted non-native tree species *Pinus radiata* D.Don were planted. As a control an unplanted plot was also installed, which will be left with no management.

Tree mortality at the Waipori was high within the first two years after planting and this trial site was abandoned. Across the remaining three sites and within the first two years *Nothofagus fusca* showed very poor survival and dead trees were replaced with *Leptospermum scoparium* (mānuka) seedlings in 2015 at Mangatoa and Rewanui and interplanted at Te Apiti (Table 2).

Tree spacing at planting was species specific (Table 2) with most species, except *Pinus radiata* and *Fraxinus excelsior* planted at 2.2 m x 2.2 m spacing. *Pinus radiata* and *Fraxinus excelsior* were planted at a slightly wider spacing of 2.8 m x 2.8 m. The location of each tree inside the measurement plot was mapped as a bearing and distance from the plot centre. Site preparation for planting consisted of initial mob grazing, followed by spot spraying.

### 4.4 Time zero assessments

Time zero assessments were undertaken in 2013, 1–2 months before planting the trees, at all sites and included a plant diversity assessment, and soil sampling and testing for chemical analysis, bulk density, porosity determination, and profile description.

### 4.5 Time zero plant diversity assessment

Vegetation composition was assessed for each 50×50 m square plot for plant species presence and relative cover. Plant species presence was recorded in height tiers [5] and relative cover for each plant species in each tier was visually estimated using a cover-class scale [6]. All plants were identified were possible to species level.

### 4.6 Time zero soil sampling, preparation and testing

Mineral soil chemistry samples were collected from 30 bulked locations per plot on a grid for each sampling depth, 0–10, 10–20, 20–30 cm depth, within the tree measurement area using a stainless steel 25 mm diameter Hoffer tube sampler. One soil bulk density sample per plot, 0–10, 10–20, 20–30 cm depth, was collected from the plot centre using a stainless steel 98 mm internal diameter core. Soil porosity samples were collected in the middle of the 0–10 cm depth using 51 mm or 54 mm internal diameter by 30 mm length cores taken in replicate, with triplicates taken at Waipouri. At the plot centre the topsoil horizon depth was recorded and a brief soil profile description [7] was visually undertaken using a Dutch auger to 1 m depth.

For each plot a sub-sample of the field fresh soil chemistry sample was sieved to <2 mm and used to examine the range and relative rates of carbon substrate utilisation by the soil microbial population present in the sub-sample. Five g of this material was placed into sterile flasks containing 100 ml of autoclaved distilled double deionized water. The flasks were sealed and agitated on an orbital shaker at 150 rpm for 45 min. After settling, 100 μl of the suspension was aseptically inoculated into a set of 32 wells on a BIOLOG Ecoplate (BIOLOG Inc., Hayward, CA, USA) [8]. Different carbon substrates were present in 31 of the wells, and the remainder was an empty control. Plates were incubated at 12 °C. Use of the carbon substrates was indicated by a colour change (measured daily at 595 nm) in tetrazolium dye that responded to respiration [9]. The data collected after 120 h incubation was selected for analysis based on the activity criteria outlined by Zak et al. [10].

Mineral soil chemistry samples were air-dried (<40 °C), sieved to retain the <2 mm mineral soil fractions and archived. The soil samples (<2 mm fraction) from three sites (FR531/1, FR531/2, and FR531/1) were tested for total carbon and total nitrogen using a LECO FPS-21,000 CNS thermal combustion furnace and reported on an oven-dry (104 °C) basis. Soil bulk density sample mineral soil fractions (<2 mm and >2 mm) and >2 mm organic matter fractions were oven dried at 104 °C and weighed. Soil bulk density was calculated using the soil mass (<2 mm and >2 mm mineral fraction) and sampling core volume. Soil porosity samples were tested for total porosity using a measure of sample bulk density and particle density, and field air capacity (% by volume of air-filled voids at 10 kPa matric potential) and macro-porosity (% by volume of pores at 5 kPa matric potential) measured by moisture release in a pressure chamber.

Diffuse reflectance MIR spectra were acquired for all soil samples <2 mm fraction using methods described in Garrett et al. [11]. In summary, sample preparation included grinding a 10 g sub-sample for 180 s in a 45 ml zirconia ceramic grinding vial containing two 12.7 mm zirconia ceramic balls using a Spex800D mixer mill. Diffuse reflectance MIR spectra were acquired for all samples, using a Bruker Invenio-S Fourier transform infrared (FTIR) spectrometer with a Bruker HTS-XT (High Throughput Screening Extension) microplate reader fitted with a liquid nitrogen cooled mercury cadmium telluride detector. The instrument system was purged with CO_2_-free dry air using a Peak Scientific PG14L generator. Sample spectra were acquired in diffuse reflectance mode with 50 co-added scans from 8000 to 400 cm^−1^ at 4 cm^−1^ resolution, using four replicate samples. The spectra were pre-processed using Bruker OPUS 8.2 software applying the make scalar compatible and cut functions to truncate the wavenumber range to 4000 to 600 cm^−1^. Soil property predictions can be generated using the average of hte four replicate spectra for New Zealand planted forest specific predictions [12] and predictions available through the USDA National Soil Survey Center–Kellogg Soil Survey Laboratory (NSSC KSSL) FT-MIR spectral library [12,13].

## Limitations

The partial establishment failure of *nothofagus fusca* limits the future comparative analysis of growth and performance of this tree species. Due to poor tree establishment the southernmost site (Waipori) was abandoned after initial data was collected.

## Ethics Statement

The authors have read and follow the ethical requirements for publication in Data in Brief and confirming that the current work does not involve human subjects, animal experiments, or any data collected from social media platforms.

## CRediT authorship contribution statement

**Thomas S.H. Paul:** Conceptualization, Methodology, Investigation, Data curation, Writing – review & editing. **Loretta G. Garrett:** Methodology, Investigation, Data curation, Writing – original draft, Writing – review & editing. **Simeon J. Smaill:** Methodology, Investigation, Data curation, Writing – review & editing.

## Data Availability

Paul, T.S.H., L.G. Garrett, and S.J. Smaill (2024). Raw data on initial soil, plant and trial details for the New Forest trial series (FR531), afforestation using a range of tree species, in New Zealand (Original data) (figshare)
